# Bibliometric analysis of limb replantation and transplantation: global research trends and hotspots (2001–2025)

**DOI:** 10.1097/MS9.0000000000005408

**Published:** 2026-07-24

**Authors:** Rongji Zhang, Li Han, Ji Shi, Meng Xiangyue, Jianzheng Zhang

**Affiliations:** a Department of Microsurgery, Fourth Medical Center of the General Hospital of the Chinese PLA, Beijing, China; b Graduate Division, Hebei North University, Zhangjiakou, China; c Hand Surgery Branch, The Fifth Hospital of Harbin City, Harbin, China

**Keywords:** amputation, bibliometric, limb, replantation, transplantation

## Abstract

This bibliometric study analyzed the fields of limb replantation and transplantation to characterize international research trends over the past 25 years. Data were retrieved from the Web of Science Core Collection, and 780 articles published between January 2001 and July 2025 were evaluated. Bibliometric tools, including VOSviewer, bibliometrix, and CiteSpace, were used to examine publication outputs, journals, authors, institutions, countries, references, keywords, and thematic maps, as well as to construct co-authorship, co-occurrence, and co-citation networks. The annual number of publications demonstrated a fluctuating but upward trend, peaking in 2020. *Plastic and Reconstructive Surgery* was the most productive and influential journal, while the United States, the University of Michigan, and Kevin C. Chung ranked highest among the contributing countries, institutions, and authors, respectively. Keyword analysis identified five major clusters: vascular reconstruction techniques, anastomosis techniques, replantation survival, complications and management in limb transplantation, tissue defect repair, and functional recovery after limb salvage. These clusters highlight both the foundations and the emerging frontiers of the field. These findings suggest that vascular reconstruction and tissue repair remain central components, while complication management and technical optimization of anastomosis represent key ongoing trends. Research on functional recovery emphasizes long-term prognosis and quality of life, and future directions may be guided by advances in tissue engineering, immunomodulation, and interdisciplinary collaborations.

## Introduction

Globally, the incidence and prevalence of traumatic limb amputations have shown a significant upward trend since 1990. According to the Global Burden of Disease database, the number of traumatic limb amputees increased from 11.37 million cases and 370.25 million people in the year of study to 13.23 million cases and 552.45 million people in 2019, representing an increase of 16.4% and 49.2%, respectively. This trend indicates a concerning situation^[^1^]^. In the military field, combat injuries predominantly affect the limbs, often leading to traumatic limb amputations[2]. During the Iraq and Afghanistan wars, approximately 30 000 soldiers sustained limb injuries due to combat, accounting for more than 75% of all war-related injuries^[^3,4^]^. Despite significant advancements in modern prosthetic technology, they still cannot fully replicate the complex functions and sensory experiences of natural limbs. Heavy psychological and economic burdens pose serious threats to individual health, family well-being, and even social harmony^[^5^]^. To avoid permanent amputation, replantation and transplantation have become the preferred treatments for traumatic limb amputation. Since the first successful replantation of a severed arm in the 1960s, limb replantation and transplantation have evolved from a simple, groundbreaking surgical technique to an integrated frontier research direction combining microsurgery, transplant immunology, biomaterials, regenerative medicine, and rehabilitation medicine^[^6^]^. The development of this field profoundly affects the quality of life of patients with severe limb amputation injuries. The dynamics of its research and the evolution of knowledge reflect advances in medical technology as well as the deeper logic of interdisciplinary integration from a new perspective.

HIGHLIGHTS

- This bibliometric study provides the first analysis of publications on limb replantation and transplantation.
- Vascular reconstruction and tissue repair were identified as key technical foundations.
- Prevention of complications and improved anastomosis techniques are major research trends.
- Functional recovery studies focus on long-term prognosis and patient quality of life.
- Advances in tissue engineering and immunomodulation may guide future clinical practice.

Bibliometrics, as a quantitative analysis method for studying the structure, distribution, and evolution patterns of scientific literature, provides a powerful and objective basis for revealing the knowledge foundation, research hotspots, core author groups, institutional collaboration networks, and future development trends in a specific academic field^[^7,8^]^. This research team conducted a bibliometric analysis to characterize the current status of limb replantation and transplantation research and explored its developmental trends, aiming to identify current hotspots in this field and provide a perspective for future research directions. This study is based on literature data from the Web of Science Core Collection (WoSCC) from 2001 to 2025, analyzing dimensions such as annual publication volume, countries/regions, research institutions, authors, source journals, keyword co-occurrence, and co-citation of references. Building on this, the study conducts an in-depth discussion of the key sub-research areas identified through bibliometric characteristics. Despite decades of progress, a systematic bibliometric overview of the research on limb replantation and transplantation is lacking. Here, we provide the first comprehensive mapping of the field, identifying its developmental trajectory and emerging research fronts. This research will help emerging scholars and field experts to better understand the research landscape, identify emerging topics, and provide a reference for shaping future research directions in limb replantation and transplantation. In addition, transparent reporting of research involving artificial intelligence is essential. This study adhered to the TITAN 2025 guidelines for the declaration and use of AI^[^9^]^.

## Materials and methods

### Data collection and retrieval strategy

**Figure 1.**
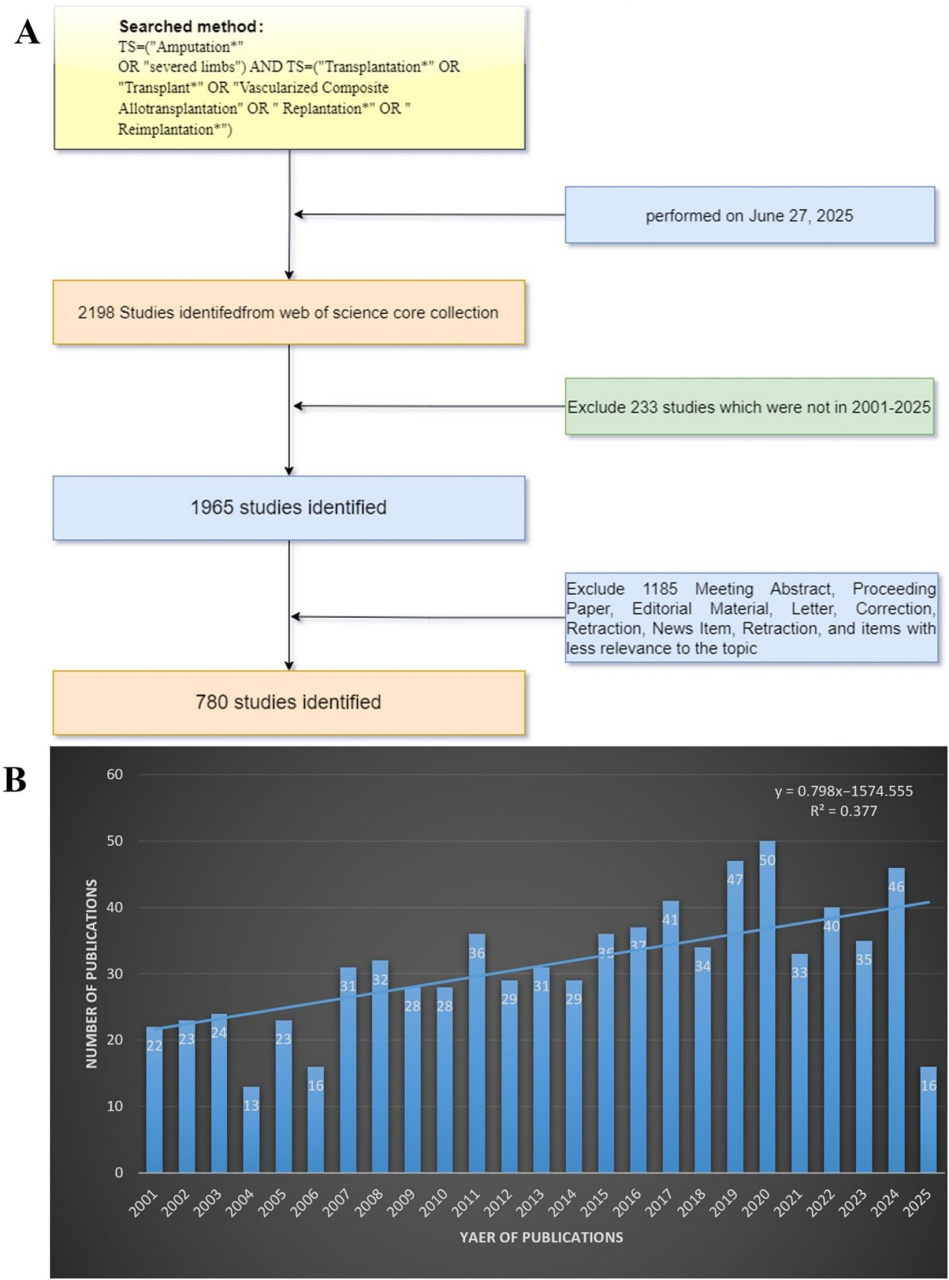
(A) Flowchart of the study selection process for bibliometric analysis. Searched method: The search was performed using terms related to limb replantation and transplantation. 2198 studies identified: Initial total number of studies found in the WoSCC. 1965 studies identified: Number of studies after excluding studies not published between 2001 and 2025. 780 studies identified: Final selected studies after excluding meeting abstracts, proceeding papers, and other irrelevant items. (B) Annual publication trend on limb replantation and transplantation from 2001 to 2025*. The bars represent the number of publications per year. The linear model fitted to the data is *Y* = 0.798*X* − 1574.555 (*R*^2^ = 0.377, *P* < 0.01). *Data for 2025 are incomplete, covering publications up to 23 July 2025.

In this cross-sectional study, publication data were collected on a single day (23 July 2025) and downloaded in “plain text” format from WoSCC. The data collection and retrieval strategies are shown in Figure 1A (retrieval flowchart). The retrieved publications met the following criteria.

1. The search terms were defined by TS (“topic,” including title, abstract, author keywords, and keywords), with the retrieval query being: TS = (“Amputation*” OR “severed limbs”) AND TS = (“Transplantation*” OR “Transplant*” OR “Vascularized Composite Allotransplantation” OR “Replantation*” OR “Reimplantation*”).
2. The document type was an article or a review article.
3. The publication period was from 1 January 2001 to 23 July 2025.
4. The following information was collected: publication, author, country, institution, journal, keywords, and citations.
5. Information collected: publications, authors, countries, institutions, journals, keywords, and citations.

Deduplication using DOI and title, with manual verification of author and institution variants (e.g., KU Leuven/Katholieke Universiteit Leuven).

To complement the WoSCC-based bibliometric analysis, a supplementary PubMed search was performed to identify clinically oriented studies, particularly clinical trials/studies in limb replantation and transplantation. The search strategy used in PubMed was: (“limb replantation”[Title/Abstract] OR “limb reimplantation”[Title/Abstract] OR “limb transplantation”[Title/Abstract] OR “extremity replantation”[Title/Abstract] OR “extremity transplantation”[Title/Abstract] OR “hand replantation”[Title/Abstract] OR “hand reimplantation”[Title/Abstract] OR “hand transplantation”[Title/Abstract] OR “finger replantation”[Title/Abstract] OR “finger reimplantation”[Title/Abstract] OR “finger transplantation”[Title/Abstract] OR “digit replantation”[Title/Abstract] OR “digit transplantation”[Title/Abstract] OR “vascularized composite allotransplantation”[Title/Abstract] OR “amputation replantation”[Title/Abstract]). This search identified 19 clinical trial records, of which 14 unique trials were retained after excluding studies that overlapped with the WoSCC dataset. These records were analyzed separately to further characterize the clinical and translational research focus in the field.

To ensure thematic relevance, duplicate records were removed from the retrieved results, and the remaining records were manually screened prior to bibliometric analysis. Titles and abstracts were reviewed, and the full texts were further assessed when necessary. Publications that were not directly related to limb replantation, limb transplantation, reconstructive microsurgery, or postoperative functional recovery were excluded.

### Data analysis and network mapping

Bibliometrics first emerged in the early 20th century and is now widely used in literature analysis^[^10^]^. Bibliometric analysis provides a quantitative method for reviewing and investigating the existing literature in a specific field^[^7^]^. We searched for relevant publications in the Science Citation Index Expanded of the Science Core Collection.

The retrieved data were analyzed using CiteSpace (version 6.4.R2)^[^11^]^ to generate visual knowledge maps. The main parameters for CiteSpace were set as follows: The time span was set from January 2001 to July 2025, with time slices set to 1 year per slice. For network generation, the node types selected included “Country,” “Institution,” “Author,” “Cited Reference,” and “Keyword” for different analyses. The selection criteria were set to “Top N = 50” per slice, meaning that the top 50 most cited or frequent items from each slice were selected for network construction. To simplify the network and highlight the most significant structural connections, the “Pathfinder” and “Pruning Sliced Networks” algorithms were applied. For the cluster analysis of keywords and references, cluster labels were generated using the Log-Likelihood Ratio algorithm to ensure the representative naming of each thematic group. In addition to CiteSpace, VOSviewer (version 1.6.20)^[^12^]^ and Excel were used to further visualize and analyze the data. During the analysis, detailed information such as authors, keywords, journals, countries, institutions, and references was retrieved and subjected to co-analysis^[^13^]^. Graphical and visual results revealed collaboration patterns with the aid of modern computer technology. This has facilitated researchers in discovering and identifying the knowledge base of a discipline while also uncovering intrinsic connections between these pieces of information, such as common research topics among different authors, the research focuses of different institutions, and new theories emerging from existing institutions^[^14^]^.

## Results

### Global annual publication output of limb replantation and transplantation

From 1 January 2001 to 23 July 2025, 780 publications related to limb replantation and transplantation were retrieved from the WoSCC. The annual publication volume is shown in Figure 1B. Overall, annual output increased over time, although this pattern was accompanied by substantial fluctuations. The lowest publication volume was observed in 2004 (*n* = 13), and the highest was recorded in 2020 (*n* = 50). Linear regression analysis showed a statistically significant relationship between annual publication volume and year (*P* < 0.01), with a regression equation *Y* = 0.798*X* − 1574.555 and an *R*^2^ value of 0.377. This *R*^2^ value indicated that the linear model had limited explanatory power for the observed temporal pattern.

### Journal contribution analysis

**Figure 2.**
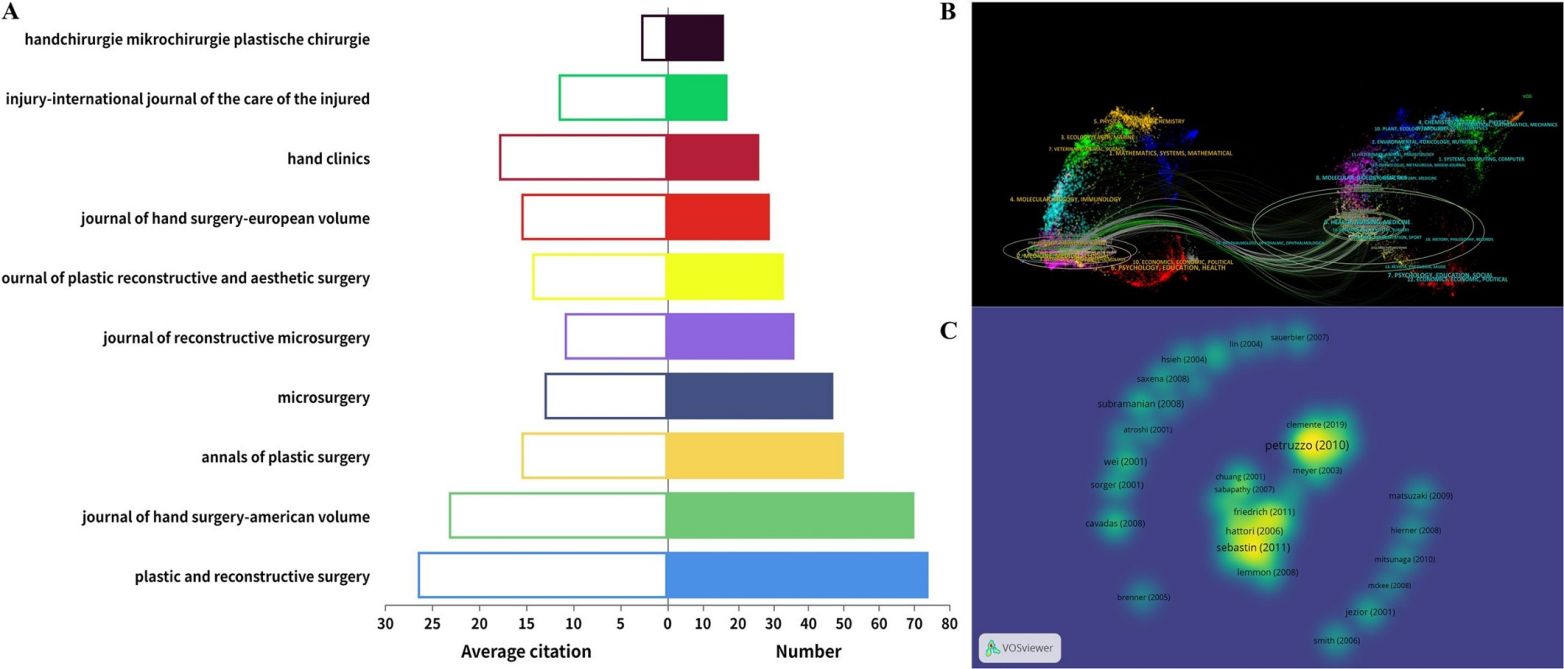
(A) Double overlay map of journals in limb replantation and transplantation research. The map shows citing journals on the left and cited journals on the right, with citation paths represented by colored lines. Each node represents a subject category, and colors indicate different clusters of disciplines. (B) Bar chart illustrating the relationship between the number of publications and average citations for top journals in limb replantation and transplantation research. Each bar represents a journal, with the length of the bar indicating the number of publications and the color corresponding to each journal. (C) Citation heatmap of influential papers in limb replantation and transplantation research. Each point represents a key study, with the size of the point corresponding to the number of citations. The color gradient from green to yellow indicates citation frequency, with yellow representing higher citation counts.

To display the distribution of citing and cited journals, we used a CiteSpace dual-map overlay. In Figure 2A, citing journals are represented on the left map, while cited journals are shown on the right map. The labels for the different disciplines are distinguished by the color hues of the lines. Citing journals primarily involve fields such as medicine, clinical studies, surgery, molecular biology, and immunology, whereas the cited journals are concentrated in disciplines such as medicine, surgery, rehabilitation, nursing, and sports. A total of 186 journals have published articles or reviews in this field. The top 10 journals with the highest output are listed in Table 1, accounting for 51% of the total publication volume (398/780). Figure 2B displays the average citation count and total number of publications in these journals in the field. Among the top 10 ranked journals, most belong to the Journal Citation Reports (JCR) Q1 and Q2 categories. *Plastic and Reconstructive Surgery* was the journal with the highest number of publications and citations.

**Table 1:** Top 10 journals by publication rank in limb replantation and transplantation.

Rank	Source	Number	Citations	Average citation	JCR	Impact factor (IF) (2024)
1	*Plastic and Reconstructive Surgery*	74	1966	26.57	Q1	3.4
2	*Journal of Hand Surgery-American Volume*	70	1628	23.26	Q2	2.1
3	*Annals of Plastic Surgery*	50	777	15.54	Q2	1.6
4	*Microsurgery*	47	615	13.09	Q2	1.7
5	*Journal of Reconstructive Microsurgery*	36	394	10.94	Q2	2.3
6	*Journal of Plastic Reconstructive and Aesthetic Surgery*	33	475	14.39	Q1	2.4
7	*Journal of Hand Surgery-European Volume*	29	451	15.55	Q2	1.6
8	*Hand Clinics*	26	466	17.92	Q3	1.1
9	*Injury-International Journal of the Care of the Injured*	17	197	11.59	Q2	2.0
10	*Handchirurgie Mikrochirurgie Plastische Chirurgie*	16	45	2.81	Q4	0.6

### Highly cited article analysis

Based on citation data from the original research articles, 780 publications received 12 320 citations, with a median citation count of 8. Table 2 lists the top 10 highly cited articles in the field of replantation and transplantation, ranging from 107 to 307 citations. Among these, the article “The International Registry on Hand and Composite Tissue Transplantation,” published in *Transplantation* in 2010, has the highest citation count (307 citations), demonstrating significant academic influence within the field. To reduce the influence of publication time on citation-based evaluation, this study calculated the Average Citations per Year (ACY) for the top 10 most-cited publications to enable a time-adjusted comparison. The supplementary analysis showed that even after accounting for differences in citation accumulation time, “The International Registry on Hand and Composite Tissue Transplantation” remained the most outstanding and representative publication.

**Table 2:** Top 10 most cited articles.

Rank	Year	First author	Title	Source	Citations	ACY
1	2010	Palmina Petruzzo	The International Registry on Hand and Composite Tissue Transplantation	*Transplantation*	307	19.19
2	2011	Sandeep J Sebastin	A systematic review of the outcomes of replantation of distal digital amputation	*Plastic and Reconstructive Surgery*	192	12.8
3	2003	Jean Kanitakis	Clinicopathologic features of graft rejection of the first human hand allograft	*Transplantation*	157	6.83
4	2001	F C Wei	The outcome of failed free flaps in head and neck and extremity reconstruction: what is next in the reconstructive ladder?	*Plastic and Reconstructive Surgery*	130	5.2
5	2006	Yasunori Hattori	A retrospective study of functional outcomes after successful replantation versus amputation closure for single fingertip amputations	*The Journal of Hand Surgery – American Volume*	128	6.4
6	2003	Jean Michel Dubernard	Functional results of the first human double-hand transplantation	*Annals of Surgery*	121	5.26
7	2007	Marco Lanzetta	Second report (1998–2006) of the International Registry of Hand and Composite Tissue Transplantation	*Transplant Immunology*	121	6.37
8	2005	Marco Lanzetta	The International Registry on Hand and Composite Tissue Transplantation	*Transplantation*	110	5.24
9	2008	Anuradha Subramanian	A decade’s experience with temporary intravascular shunts at a civilian level I trauma center	*Journal of Trauma and Acute Care Surgery*	109	6.06
10	2011	Jeffrey B Friedrich	Epidemiology of upper extremity replantation surgery in the United States	*The Journal of Hand Surgery – American Volume*	107	7.13

Additionally, 120 publications each received more than 30 citations, indicating that a substantial number of highly influential core publications have emerged within this research domain (Fig. 2C).

### Country/region distribution and co-authorship

**Figure 3.**
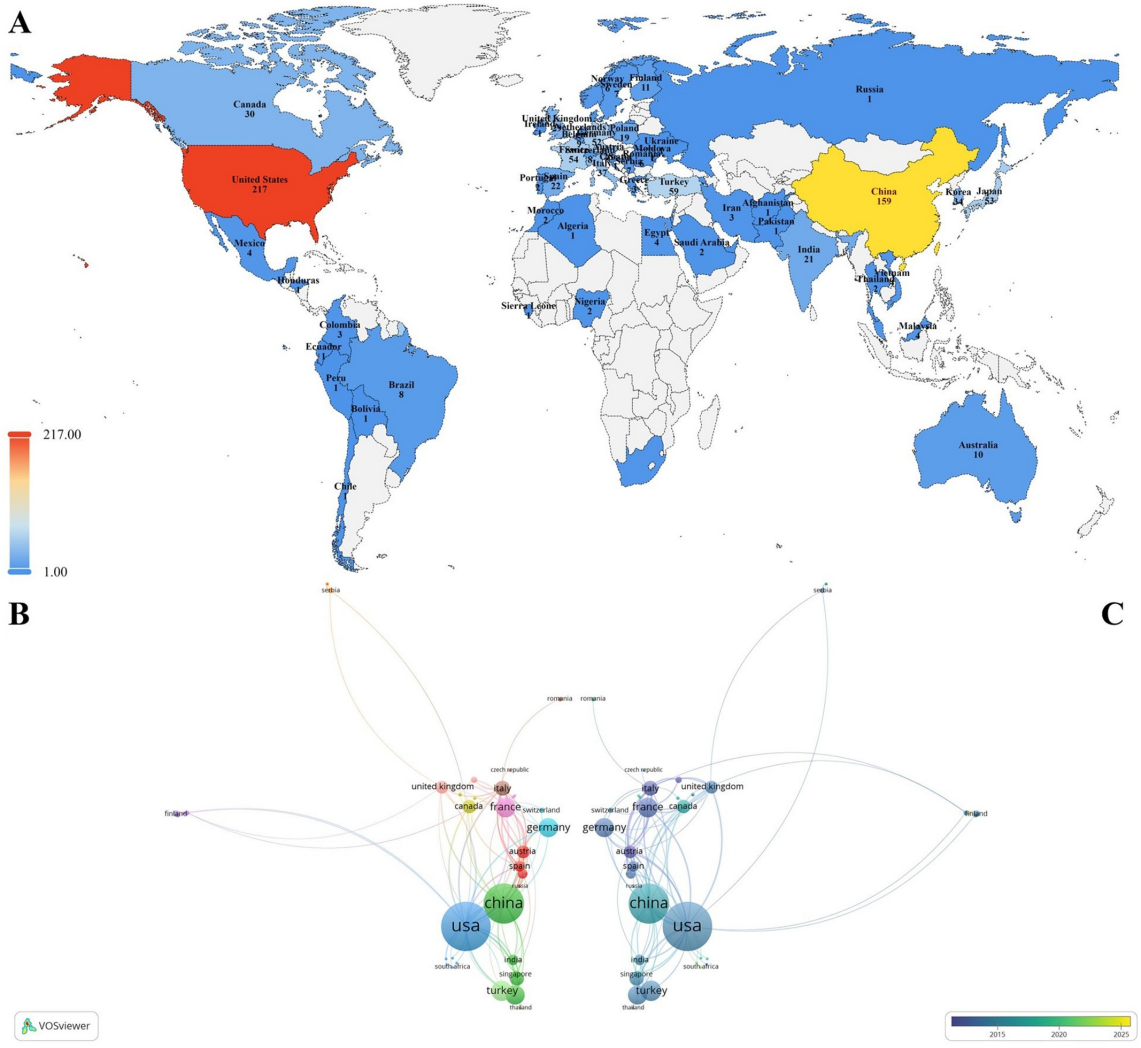
Network visualization of international collaboration in limb replantation and transplantation research. (A) Each node represents a country, and the size of the node corresponds to the total number of publications from that country. The edges between nodes indicate collaborative relationships between countries. (B) The color gradient, ranging from blue to yellow, represents the time span of publications, with blue indicating earlier publications and yellow indicating more recent ones. (C) Global distribution of publications on limb replantation and transplantation research. The map illustrates the number of publications from each country, with darker red colors indicating higher publication volumes and blue indicating lower publication volumes.

A total of 60 countries/regions worldwide have contributed to research on limb replantation and transplantation (Fig. 3A). Table 3 lists the top 10 countries/regions in terms of publication output. The United States was the leading contributor, with 217 publications and 4989 citations, followed by China, with 159 publications and 2061 citations. To examine international collaboration further, VOSviewer was used to construct a co-authorship network of countries/regions. Among the 60 countries/regions, 48 met the threshold for inclusion in the co-authorship analysis and were grouped into 11 clusters (Fig. 3B). The United States had the largest number of collaborative partners (*n* = 26), followed by China (*n* = 16). The overlay visualization further indicated that China has participated in an increasing number of collaborative studies in recent years (Fig. 3C).

**Table 3:** Top 10 productive countries/regions.

Rank	Country	Publications	Citations	Average citation
1	United States	217	4989	22.99
2	China	159	2061	12.96
3	Turkey	59	598	10.14
4	France	54	1489	27.57
5	Japan	53	918	17.32
6	Germany	52	749	14.40
7	Italy	37	1545	41.76
8	Korea	34	515	15.15
9	Canada	30	444	14.80
10	Austria	28	1161	41.46

### Distribution and co-authorship of institutions

**Figure 4.**
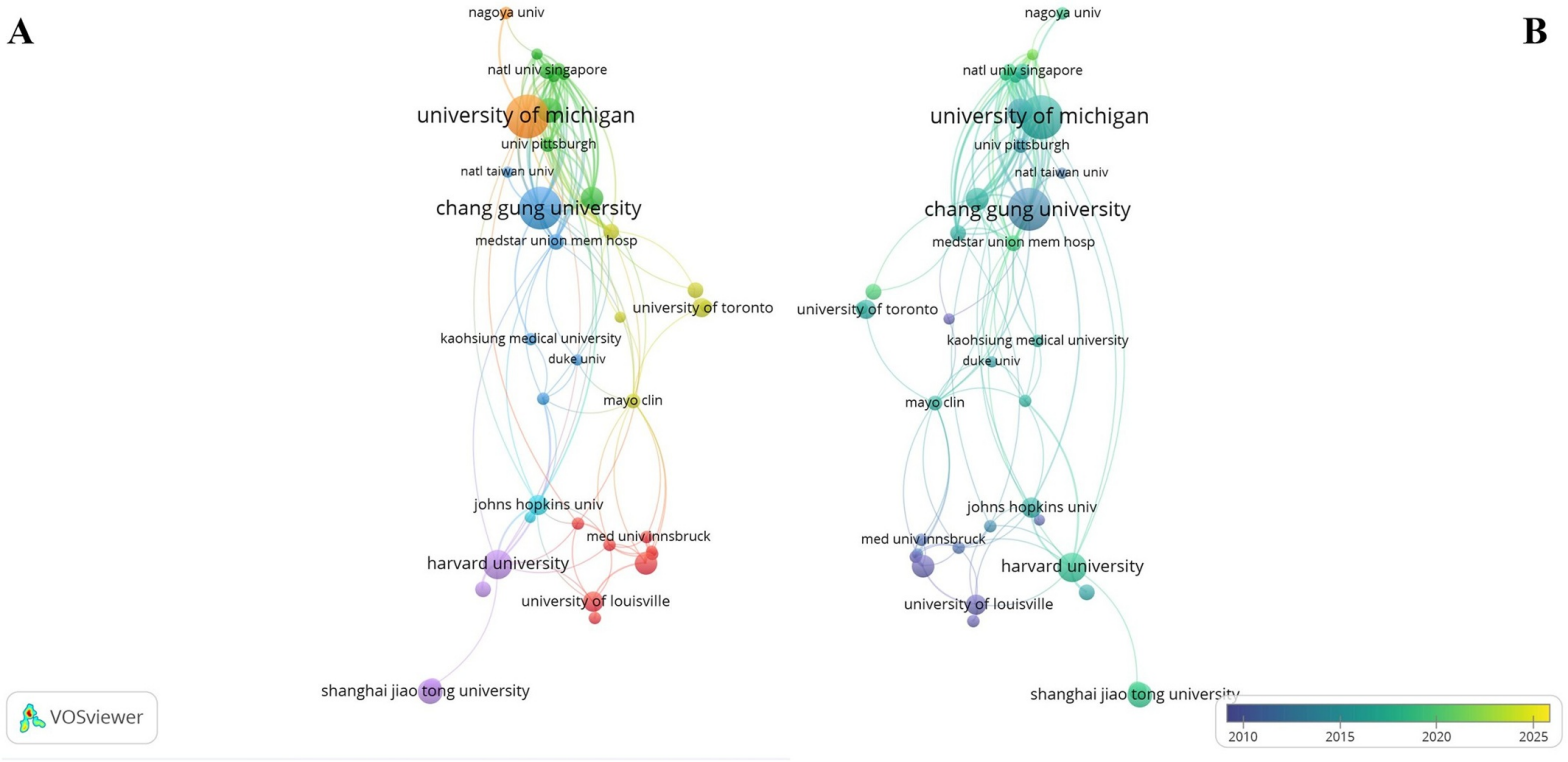
Visualization of institutional collaboration in limb replantation and transplantation research. (A) The network graph illustrates the collaborative relationships between different institutions. Each node represents an institution, and the connections between them indicate collaborative publications. (B) A time-based evolution of the collaborations from 2010 to 2025. The color gradient from blue to yellow represents the timeline of collaborations, with blue indicating earlier collaborations and yellow indicating more recent ones. The graph shows the progression of collaborative ties.

A total of 978 institutions participated in this study on limb replantation and transplantation. The top 10 most productive institutions are listed in Table 4. The University of Michigan published 41 papers, cited 1169 times, followed by Chang Gung University (38 papers, cited 779 times) and Harvard University (22 papers, cited 372 times). Using a minimum threshold of five publications per institution, 52 institutions met the criteria. VOSviewer was employed to conduct a co-authorship analysis of the 52 highly productive institutions. The collaboration network of the institutions is shown in Figure 4A. The co-authorship network of 36 institutions was divided into 7 clusters, each represented by a different color. In recent years, the influence of US institutions in the field of limb replantation and transplantation has grown significantly, as clearly demonstrated by overlay visualization (Fig. 4B).

**Table 4:** Top 10 institutions by productivity.

Rank	Organization	Country	Publications	Citations
1	University of Michigan	United States	41	1169
2	Chang Gung University	China	38	779
3	Harvard University	United States	22	372
4	Ganga Hospital	India	16	298
5	Shanghai Jiao Tong University	China	16	143
6	Edouard Herriot Hospital	France	14	993
7	University of Washington	United States	14	395
8	University of Louisville	United States	12	343
9	Johns Hopkins University	United States	12	183
10	University of Toronto	Canada	11	146

### Distribution and co-authorship of authors

**Figure 5.**
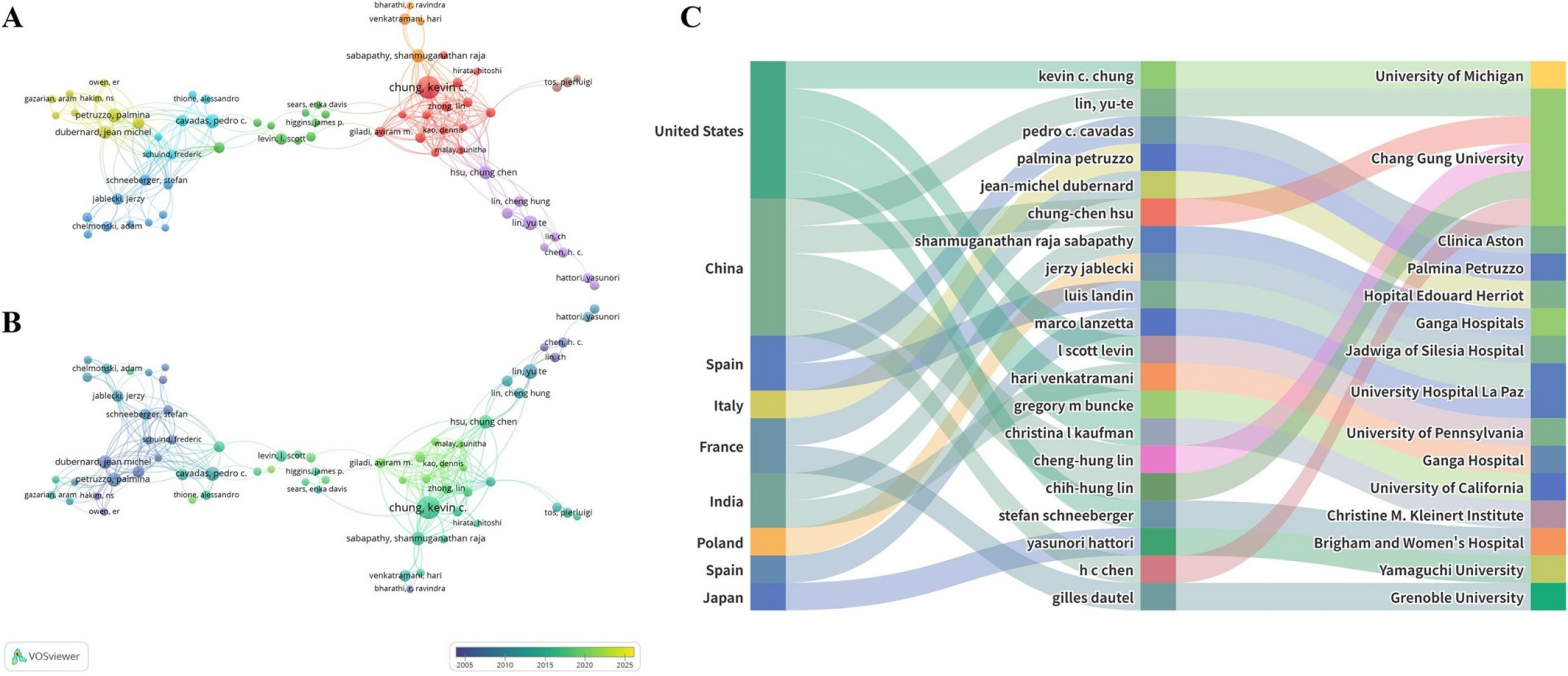
Visualization of the author co-authorship network and its temporal overlay in limb replantation and transplantation research. (A) Co-authorship network, where each node represents an author, and node size corresponds to the number of publications. Links between nodes indicate co-authorship relationships. Different colors represent distinct clusters of collaborative groups. (B) Temporal overlay of co-authorship, where the color gradient from blue to yellow indicates the average publication year, with blue representing earlier collaborations and yellow representing more recent ones. (C) Sankey diagram illustrating the collaboration between countries and key authors in limb replantation and transplantation research. Each node represents either a country or an author, and the flow lines between the nodes indicate collaborations between them. The color of the flow lines corresponds to the country of the author, and the width of the line represents the strength or frequency of the collaboration. The United States and China are major contributors, with authors such as Kevin C. Chung and Yu-Te Lin central to many collaborations.

A total of 780 articles and 3091 authors were included in the co-authorship network analysis of limb replantation, with an average of 4 authors per article. Visual analysis of these data was conducted using the VOSviewer software to identify prolific authors, collaboration patterns, and research clusters, providing valuable insights into the collaborative structure and research trends in this field. Table 5 lists the top 10 productive authors. Among them, Kevin C. Chung was the most prolific author, with 35 publications and 961 citations, followed by Yu-Te Lin (15 publications, 330 citations) and Palmina Petruzzo (13 publications, 1149 citations). When analyzing the co-authorship network using VOSviewer, a minimum publication threshold of four articles per author was set, resulting in the inclusion of 101 authors. A co-authorship analysis was conducted with the 101 authors using VOSviewer. The co-authorship network of the authors is shown in Figure 5A. The collaboration network, consisting of 67 authors, was divided into 8 clusters, each represented by a different color. Hsu had the highest number of connections in the co-authorship network, with 19 collaborators, followed by Chung, with 18. Among the top 10 most productive authors, Spain contributed the most (3 authors), followed by China (2 authors). The overlay visualization (Fig. 5B) indicates the formation of a new research group centered on the Chinese scholar Kevin C. Chung. Figure 5C illustrates the relationships among the top 20 authors, their countries, and affiliated institutions.

**Table 5:** Top 10 productive authors.

Rank	Author	Country	Publications	Citations	Average citation
1	Kevin C. Chung	United States	35	961	27
2	Yu-Te Lin	China	15	330	22
3	Palmina Petruzzo	Italy	13	1149	89
4	Pedro C. Cavadas	Spain	13	615	47
5	Jean-Michel Dubernard	France	12	1115	93
6	Shanmuganathan Raja Sabapathy	India	12	245	20
7	Chung-Chen Hsu	China	12	241	20
8	Marco Lanzetta	Spain	9	908	101
9	Luis Landin	Spain	9	533	59
10	Jerzy Jablecki	Poland	9	531	59

### High-frequency keyword co-occurrence analysis

**Figure 6.**
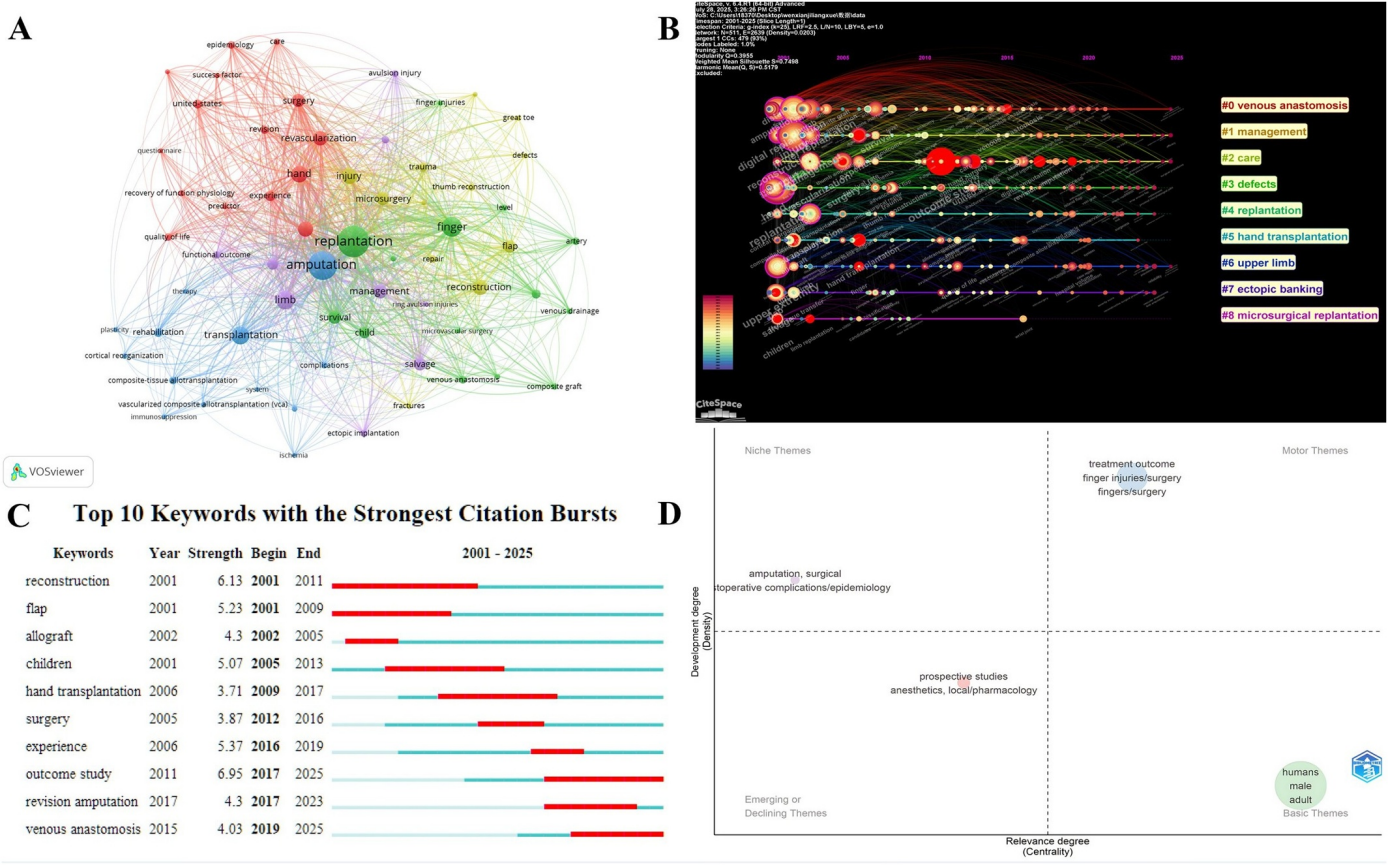
(A) Citation heatmap of key publications in limb replantation and transplantation research. Each point represents a citation for a specific paper, with the size of the point corresponding to the number of citations. The color gradient, ranging from blue to yellow, indicates citation frequency, with yellow representing the highest citation counts. (B) Keyword evolution and trend analysis in limb replantation and transplantation research from 2001 to 2025. Each node represents a keyword, with the size of the node corresponding to the frequency of that keyword in the literature. The connections between the nodes indicate the relationships between keywords over time, with the timeline showing the evolution of trends from 2001 to 2025. (C) Top 10 keywords with the strongest citation bursts in limb replantation and transplantation research (2001–2025). Each keyword is displayed with its citation burst strength, along with the start and end years of the burst period. The red bar indicates the citation burst period, with the length of the bar representing the strength of the citation burst. (D) Thematic map of PubMed-indexed clinical studies/trials related to limb replantation and transplantation. The upper-right quadrant represents motor themes, the upper-left quadrant niche themes, the lower-right quadrant basic themes, and the lower-left quadrant emerging or declining themes.

The keywords covered the main themes of the publications, making high-frequency keywords well-suited for co-occurrence analysis. VOSviewer was used to extract and cluster keywords that appeared 10 times or more in this study, with 63 keywords in total (Fig. 6A). The 63 high-frequency keywords were grouped into 5 clusters. We analyzed the five main clusters as follows:

1. Cluster 1 (red): Vascular reconstruction techniques in replantation and transplantation, including vascular reconstruction, surgery, and outcomes.
2. Cluster 2 (green): Impact of anastomosis on the survival rate of finger replantation in children, including fingers, children, survival rates, and anastomosis.
3. Cluster 3 (blue): Treatment of limb transplantation complications, including skeletal muscle, ischemia, immunosuppression, and other complications.
4. Cluster 4 (yellow): Repair and reconstruction of tissue defects, including reconstruction, defects, flaps, and free tissue transplantation.
5. Cluster 5 (purple): Functional recovery after limb salvage, including management, function, follow-up studies, and salvage.

The timeline images show the appearance period and trends of different research keywords. According to Figure 6B, “venous anastomosis,” “management,” “nursing,” and “defects” have been continuous research hotspots since 2001, with long-term attention from researchers. Burst word analysis can reveal research hotspots during specific periods. Figure 6C shows the top 10 keywords with the highest citation strength.

### Supplementary analysis of PubMed-indexed clinical literature

A supplementary thematic map based on PubMed clinical studies/trials was generated to further identify the major focus of clinically oriented research on limb replantation and transplantation (Fig. 6D). The theme “treatment outcome/finger injuries/surgery/fingers/surgery” was located in the upper-right quadrant, indicating a motor theme with both high centrality and density. “Humans/male/adult” appeared in the lower-right quadrant, corresponding to a basic theme. In contrast, “amputation, surgical/postoperative complications/epidemiology” was distributed in the upper-left quadrant as a niche theme, whereas “prospective studies/anesthetics, local/pharmacology” was located in the lower-left quadrant, representing an emerging or less developed theme. These findings suggest that the current PubMed-indexed clinical research is mainly focused on treatment outcomes and finger-related surgical management.

## Discussion

### The global upward trend in limb replantation and transplantation research

This bibliometric analysis provides a comprehensive overview of the research landscape of limb replantation and transplantation over the past 25 years. Overall, the number of publications in this field showed an upward trend, increasing from 13 articles in 2001 to 50 in 2020, which indicates growing academic interest and sustained attention to this topic. However, the relatively low coefficient of determination (*R*^2^ = 0.377) suggests that this trend cannot be adequately explained using a simple linear growth model. Rather than reflecting a stable and continuous increase, the fluctuation in annual publications more likely mirrors the evolving maturity of the field, shifts in research priorities, and the influence of landmark clinical advances, particularly in vascularized composite allotransplantation. In addition, broader external factors, such as the COVID-19 pandemic, may also have affected surgical practice, research productivity, and international collaboration, thereby contributing to the temporal variability in publication output.

From the perspective of knowledge dissemination, limb replantation and transplantation research has attracted broad, multidisciplinary attention. The top productive journals were mainly concentrated in the fields of medicine, surgery, and transplantation, reflecting the interdisciplinary nature of this research area and its strong orientation toward clinical translation. Notably, most of the top 10 productive journals were classified as JCR Q1 or Q2, suggesting that the field has gradually improved in terms of both academic quality and scholarly influence. Among these journals, *Plastic and Reconstructive Surgery* occupied a particularly prominent position, accounting for 9.5% of the total publications and demonstrating high citation performance, which underscores its role as a core platform for disseminating research in this specialized field.

At the global level, the field has demonstrated broad international participation, with contributions from 60 countries and regions. Among them, the United States and China were the two most productive countries, indicating their central roles in shaping the research landscape. The United States ranked first in both publication output and citation impact, whereas China ranked second in publication volume. Co-authorship analysis further revealed that both countries were actively engaged in international collaboration, and frequent cooperation between them highlights their importance within the global scientific network.

The United States accounts for a dominant 50% share among the top 10 research entities, followed by China (20%), with India, France, and Canada each representing 10%. This distribution reflects well-established research networks organized around these geographic hubs. Quantitatively, US institutions maintain a leading position in limb replantation and transplantation research, as demonstrated in Table 3.

A supplementary analysis based on PubMed adds a clearer clinical dimension to the bibliometric framework derived from WoSCC. Overall, the thematic patterns identified by PubMed resonate with the overall findings from WoSCC, indicating that research in this field focuses on both technological advancements and clinical outcomes. In contrast, the PubMed data further highlight themes such as treatment outcomes and finger injuries/surgery, suggesting that current clinical research is more directly focused on surgical efficacy, postoperative recovery, and functional outcomes. These findings suggest that WoSCC is better suited for revealing the overall distribution and evolution of research hotspots, while supplementary analysis from PubMed helps clarify how these hotspots manifest in clinical practice. It should be noted that the prominence of terms such as humans, males, and adults is largely influenced by PubMed indexing and demographic tags. Therefore, these results should be interpreted with caution.

### Knowledge base hotspots and emerging frontiers in limb replantation and transplantation research

By clustering the 63 keywords that appeared more than 10 times, 5 major thematic groups were identified:

1. Cluster 1 (red): Vascular reconstruction techniques and their applications, encompassing vascular reconstruction, surgical procedures, clinical outcomes, and surgical experience.
2. Cluster 2 (green): Anastomotic techniques and replantation survival, including keywords such as fingers, children, survival rate, and anastomosis.
3. Cluster 3 (blue): Complications and treatment in limb transplantation, including transplantation, ischemia, immunosuppression, and associated complications.
4. Cluster 4 (yellow): Repair and reconstruction of tissue defects, involving reconstruction, tissue defects, flaps, and free tissue transfer.
5. Cluster 5 (purple): Functional recovery following limb salvage, with a focus on management, functional outcomes, follow-up studies, and salvage procedures.

Co-occurrence analysis of high-frequency keywords revealed the primary research directions and thematic focus within the field. Examination of both the keywords and the timeline of the top 10 burst terms demonstrates that from 2001 to 2025, topics related to vascular reconstruction techniques and functional recovery have remained at the forefront of scholarly attention. This indicates that investigations of surgical procedures and postoperative prognosis have consistently constituted central research hotspots. In recent years, research on the repair and reconstruction of tissue defects has become an increasingly significant area of inquiry. Notably, these studies are often closely integrated with prognostic analyses, reflecting a strong emphasis on long-term outcomes. This trend closely aligns with the results of our cluster analysis, thus further validating the identified research patterns. Taken together, these five clusters provide a systematic perspective on the evolution of this research field. They highlight both enduring priorities and emerging themes, thereby offering a comprehensive understanding of the current hotspots and future directions in limb replantation and transplantation research.

#### Red cluster: vascular reconstruction techniques and their applications

Vascular reconstruction is key to the success of limb replantation and transplantation surgery, directly affecting tissue blood supply and limb survival rates. The development of microsurgical techniques has increased the precision of vascular reconstructions. However, prolonged ischemia, severe vascular damage, and tissue compression caused by traumatic injuries remain the major clinical challenges. Agarwal *et al*^[^15^]^ demonstrated that the use of temporary intravascular shunts can serve as an effective method of provisional vascular reconstruction, significantly reducing ischemia time and providing a window for microsurgical repair, particularly in cases of severe ischemia.

Novel minimally invasive techniques and 3D-printed vascular scaffolds are driving innovation in vascular repair. Farzin *et al*^[^16^]^ pointed out that traditional microsutures are time-consuming and prone to thrombosis, whereas 3D-printed sugar-based scaffolds can effectively shorten anastomosis time and reduce leakage and have already demonstrated feasibility in animal experiments. Liu *et al*^[^17^]^ reported the successful application of a magnetic pin-ring device in a canine liver transplantation model, which not only significantly shortened the surgical time but also improved vascular patency and promoted healing. These new technologies suggest that future vascular reconstruction may shift from “suture-dependent” to “device-assisted surgery,” with the potential to improve surgical efficiency and consistency. In pediatric patients, small vessel diameter and vasospasm present greater technical challenges^[^18^]^. Lin *et al*^[^19^]^ found that heparinized saline hydraulic dilation effectively improved vessel diameter and blood flow, aiding in vascular reconstruction.

Continuous advances in vascular reconstruction techniques have played a significant role in improving survival and functional recovery of replanted and transplanted limbs. However, the effect of ischemic time on tissue viability remains unclear. With the development of *ex vivo* limb preservation, robot-assisted surgery, and tissue engineering technologies, surgeons may be able to overcome current limitations and achieve higher levels of repair and functional restoration. This progress not only holds promise for optimizing clinical outcomes but also for driving microsurgery toward greater precision and personalization.

#### Green cluster: anastomosis techniques and replantation survival rates

The long-term survival of replanted and transplanted limbs depends, to some extent, on the choice and execution of the anastomosis techniques. End-to-end suturing remains the standard approach, whereas vein grafting is indispensable when vascular damage is severe or when there is a significant defect. Martin *et al*^[^20^]^ suggested that vein grafts are highly valuable in vascular reconstruction, which is consistent with clinical experience. At the same time, the quality and number of vascular anastomoses are widely regarded as key factors determining success or failure^[^21,22^]^. Arterial anastomosis, as the core step for successful replantation, ensures adequate arterial blood flow to meet the metabolic demands of tissues and reduce the risk of complications. Although single-artery repair can maintain tissue viability, studies have shown that increasing the number of arterial anastomoses improves survival rates and promotes healing^[^21,23,24^]^. However, high-quality randomized controlled trials to clearly determine the advantages and disadvantages of different arterial anastomosis techniques are lacking. Therefore, a surgeon’s technical proficiency remains a decisive factor influencing surgical success.

Venous repair is closely related to arterial anastomosis and is essential for ensuring stable blood return. Adequate venous drainage can prevent venous congestion, thereby reducing complications such as edema, increased interstitial pressure, insufficient arterial perfusion, and accumulation of metabolic byproducts^[^25^]^. Shaterian *et al*^[^21^]^ noted that increasing the number of venous anastomoses helps optimize survival outcomes in digit replantation. Arterial-only replantation combined with venous drainage is a feasible treatment strategy when venous anastomosis is limited. Puhaindran *et al*^[^26^]^ reported the “skin pocket” technique, in which the epidermis of the replanted fingertip is removed, and the fingertip is secured to an adjacent finger, the palm, or the abdomen. After waiting 2–3 weeks for neovascularization to establish venous outflow, the fingertips were separated, achieving a survival rate of 85%. Arata *et al*^[^27^]^ applied the same method without arterial or venous anastomosis, with a survival rate of 81%. Purisa *et al*^[^28^]^ proposed the medullary cavity drainage method, in which a perforated needle is inserted into the medullary cavity to drain venous blood while simultaneously performing bone fixation. The survival rate of the fingertips was 88%. Koshima *et al*^[^29^]^ reported a delayed venous anastomosis technique in which arterial repair was performed first, followed by venous dilation and anastomosis 8–12 hours later, achieving a success rate of 85.7%. These studies indicate that although venous anastomosis remains the preferred option, alternative strategies can achieve high success rates when venous anastomosis is not feasible, thereby advancing distal replantation techniques. With the development of microsurgical technology, emerging anastomosis techniques, such as simultaneous anastomosis, open-loop suturing, and air knotting, have significantly shortened surgical time, reduced ischemia duration, and improved surgical efficiency^[^30,31^]^. However, it remains unclear whether these innovative methods can significantly improve replantation survival rates.

#### Blue cluster: complications and treatment in limb transplantation

Limb transplantation shows substantial potential for restoring motor and sensory functions; however, complications remain the core challenge, limiting its long-term success. Win *et al*^[^32^]^ found that acute rejection is nearly universal; approximately 85% of patients experienced at least one episode of acute skin rejection within the first year after transplantation, and up to 56% experienced multiple episodes. This underscores the unique immunological role of the skin as a “sentinel organ.” However, the mechanisms underlying chronic rejection remain unclear. Vascular lesions, fibrosis, and degenerative tissue changes often lead to irreversible functional loss, a phenomenon confirmed in clinical practice. These findings highlight the urgent need to establish standardized diagnostic criteria and early predictive markers for chronic rejection^[^33–35^]^.

In addition to rejection response, secondary complications induced by immunosuppressive therapy also pose a significant threat to patient prognosis. For example, calcineurin inhibitors (such as tacrolimus) are effective in reducing rejection rates and promoting nerve regeneration but are strongly associated with the development of new-onset diabetes and kidney dysfunction^[^36,37^]^. Therefore, achieving a balance between “rejection control” and “immune burden” has become an urgent challenge that must be addressed. Some centers have attempted innovative strategies, such as donor bone marrow cell infusion, to reduce dependence on immunosuppressive agents, but their efficacy and safety still require confirmation through large-scale clinical trials^[^38^]^. Advances in tissue engineering and regenerative medicine have provided new insights in this field. Jank *et al*^[^39^]^ employed decellularized scaffold technology to reconstruct the natural tissue architecture of limbs while simultaneously reducing immunogenicity. Such techniques have the potential to reduce or even eliminate the need for systemic immunosuppression, thereby lowering the risk of related complications and offering a safer and more sustainable solution.

Although current therapies can ensure high short-term survival rates, the long-term prognosis remains poor. Future efforts should focus on elucidating the immunological mechanisms of chronic rejection, identifying reliable biomarkers, developing personalized immune modulation strategies, and promoting the application of early immune monitoring. Only on this foundation can limb transplantation truly overcome the “high-risk, low-reward” skepticism and achieve successful translation into routine clinical practice.

#### Yellow cluster: repair and reconstruction of tissue defects

In clinical practice, limb injuries are often accompanied by varying degrees of skin and soft-tissue defects. Achieving both anatomical and functional reconstruction while restoring the blood supply has long been a key research focus in microsurgery. Soft tissue repair provides vascular coverage for the bone, supports nerve regeneration, and maximizes the restoration of both the cosmetic appearance and functional outcomes of the limb. Shieh *et al*^[^40^]^ suggested that volar and lateral V-Y advancement flaps could be used to preserve finger length and restore protective sensations in cases of digital amputation. The quality of soft tissue directly influences healing outcomes. Vascularized soft tissue coverage provides oxygen and nutrients to the fracture site and promotes bone healing by delivering growth factors and osteoprogenitor cells. Chan *et al*^[^41^]^ found that muscle flaps, with their abundant stem cells and osteogenic factors such as bone morphogenetic protein, insulin-like growth factor 1, and fibroblast growth factor 2, can accelerate the bone repair process. In clinical practice, timely soft tissue coverage is particularly crucial, with the optimal window falling between 72 hours and 1 week after injury. Interventions within this period can significantly reduce the risk of infection and create a favorable environment for bone healing. Negative pressure wound therapy (NPWT), used as an adjunctive treatment during the interim phase, has been shown to further optimize the wound bed. Lok *et al*^[^42^]^ demonstrated that NPWT helps control infection, promotes granulation tissue formation, and provides an ideal foundation for definitive soft tissue coverage. In addition, soft tissue coverage can effectively prevent infection, making it a key factor in reducing the risk of osteomyelitis and ensuring the long-term stability of skeletal reconstruction^[^43^]^.

In clinical practice, particularly in the treatment of severe limb amputations, optimizing the application of biomaterials and cell-based therapies to further enhance the repair of bone, tendons, and soft tissues will be a major focus of future research^[^44,45^]^. With the continued development and clinical translation of these technologies, more efficient and personalized treatment strategies may become available, significantly improving patient recovery outcomes and quality of life.

#### Purple cluster: functional recovery after limb salvage

Functional recovery following limb replantation and transplantation is a complex, multifactorial process that requires the integration of surgical precision, early rehabilitation, and individualized care. The overarching goal is to restore both motor and sensory functions, along with improving the overall quality of life. Achieving this outcome depends not only on the technical expertise of the surgical team but also on sustained multidisciplinary collaboration and long-term rehabilitation interventions^[^46^].^ Early rehabilitation strategies play a decisive role in shaping the long-term outcomes. Protocols such as early controlled active motion and passive range-of-motion exercises are designed to prevent tendon adhesion and joint stiffness, both of which are major barriers to functional recovery. Young *et al*^[^47^]^ demonstrated that these interventions effectively enhanced mobility restoration and reduced postoperative complications, highlighting their critical role in the early postoperative phase. Sensory recovery, although often slower than motor recovery, is crucial. Initiating sensory re-education approximately 8 weeks after surgery through exercises such as object recognition and tactile feedback training has been shown to facilitate the return of fine discriminatory sensation^[^48^]^. Beyond conventional physical and sensory rehabilitation, adjunctive measures such as occupational therapy, therapeutic exercise, and the application of customized orthotic devices contribute significantly to muscle strength, flexibility, and coordination^[^47,49^]^. Importantly, while objective measures of functional recovery vary across individuals, the evaluation of patient-reported outcomes, particularly quality of life and psychological well-being, has emerged as an indispensable dimension of postoperative success^[^50^]^. Bueno *et al*^[^51^]^ reported that despite persistent functional deficits, many patients expressed high levels of satisfaction, particularly in relation to body image and self-esteem. These findings underscore a critical insight: successful limb salvage should not be defined solely by objective motor and sensory recovery but equally by psychological adaptation and social reintegration. Thus, the trajectory of recovery must be understood as a continuum in which the surgical, rehabilitative, and psychosocial domains intersect to determine the long-term outcomes.

## Limitations

This study used bibliometric methods to analyze the evolution and trends of limb replantation and transplantation. Bibliometric analysis is relatively comprehensive and objective; however, similar to previous studies, it has some unavoidable limitations^[^52,53^]^. In this study, publications related to limb replantation and transplantation were only extracted from the WoSCC database. Although WoSCC is the most widely used and authoritative comprehensive database^[^54–56^]^, some publications were still not included. Another limitation is that the quality of publications cannot be comprehensively assessed, which means that both high- and low-quality publications are assigned equal weights. Although we conducted multiple screenings for institutions, authors, and keywords automatically extracted by VOSviewer, some discrepancies may still exist. In addition, the inclusion of publications from 2025 allowed the present study to capture the most recent developments in this field. However, as these studies are newly published, their citation counts remain temporally constrained and may not fully reflect their eventual academic impact. This limitation should be considered when interpreting citation-based indicators in recent years.

Although ACY was added as a supplementary time-adjusted indicator for the top 10 most-cited publications, more comprehensive normalized citations or quality-weighted metrics were not applied across the full dataset. Future studies could extend this analysis by incorporating normalized citation indicators for all publications. In addition, the PubMed Clinical sub-database has a small sample size, and the topic map serves only as an exploratory supplement.

## Conclusions

To the best of our knowledge, this study is the first to provide a comprehensive review of the field of severe limb replantation and transplantation from a bibliometric perspective. A visual research framework was constructed by reviewing relevant literature and illustrating publication trends, global collaboration patterns, and research hotspots in this field. According to a bibliometric analysis, *Plastic and Reconstructive Surgery* is considered the most influential journal in this field. The United States is the leading country in terms of research publications, with research from the University of Michigan being widely cited. Chung is recognized as the most influential scholar in this field.

Through keyword analysis, we identified five key research directions and their representative trends in the field of limb replantation and transplantation: vascular reconstruction techniques and tissue defect repair are foundational and core components, with ongoing advancements providing technical support for complex transplants; the prevention and management of complications have gained increasing attention, contributing to enhanced overall treatment safety and success rates; the application of various anastomosis techniques in digit replantation highlights the optimization of techniques in alignment with clinical needs; and research on functional recovery after limb salvage is driving improvements in long-term prognosis and quality of life. In the future, the synergistic promotion and integrated development of these directions will provide continuous momentum for refining comprehensive strategies for limb transplantation and replantation. These findings offer significant insights and guidance for the future development of this field.

## Data Availability

The datasets analyzed for this study can be found in the WoSCC. The raw data supporting the conclusions of this article will be made available by the authors, without undue reservation, to any qualified researcher upon reasonable request.

## Research registration unique identifying number (UIN)

Not applicable. This manuscript is a bibliometric review and does not involve a prospective interventional clinical trial; therefore, research registration was not required.

## References

[1] Yuan B Hu D Gu S. The global burden of traumatic amputation in 204 countries and territories. Front Public Health 2023;11:1258853. 37927851 10.3389/fpubh.2023.1258853 PMC10622756

[2] Penn-barwell JG Roberts SAG Midwinter MJ. Improved survival in UK combat casualties from Iraq and Afghanistan: 2003-2012. J Trauma Acute Care Surg 2015;78:1014–20. 25909424 10.1097/TA.0000000000000580

[3] Starnes BW Beekley AC Sebesta JA. Extremity vascular injuries on the battlefield: tips for surgeons deploying to war. J Trauma 2006;60:432–42. 16508513 10.1097/01.ta.0000197628.55757.de

[4] Holcomb JB McMullin NR Pearse L. Causes of death in U.S. Special operations forces in the global war on terrorism: 2001-2004. Ann Surg 2007;245:986–91. 17522526 10.1097/01.sla.0000259433.03754.98 PMC1876965

[5] Hughes W Goodall R Salciccioli JD. Editor’s choice - trends in lower extremity amputation incidence in European Union 15+ Countries 1990-2017. Eur J Vasc Endovasc Surg 2020;60:602–12. 32709465 10.1016/j.ejvs.2020.05.037

[6] Chen ZW Bao Y Qian YQ. Reimplantation of a traumatic complete amputation of the forearm (report of a successful case). Natl Med J China 1963;49:615–20.

[7] Mayr P Scharnhorst A. Scientometrics and information retrieval: weak-links revitalized. Scientometrics 2015;102:2193–99.

[8] Agarwal A Durairajanayagam D Tatagari S. Bibliometrics: tracking research impact by selecting the appropriate metrics. Asian J Androl 2016;18:296–309. 26806079 10.4103/1008-682X.171582 PMC4770502

[9] Agha R Mathew G Rashid R. Transparency in the reporting of Artificial INtelligence – the TITAN guideline. Prem J Sci 2025;10:100082.

[10] Peng C Kuang L Zhao J. Bibliometric and visualized analysis of ocular drug delivery from 2001 to 2020. J Control Release 2022;345:625–45. 35321827 10.1016/j.jconrel.2022.03.031

[11] Chen C. A glimpse of the first eight months of the COVID-19 literature on microsoft academic graph: themes, citation contexts, and uncertainties. Front Res Metr Anal 2020;5:607286. 33870064 10.3389/frma.2020.607286 PMC8025977

[12] van Eck NJ Waltman L. Software survey: vOSviewer, a computer program for bibliometric mapping. Scientometrics 2010;84:523–38. 20585380 10.1007/s11192-009-0146-3 PMC2883932

[13] Newman MEJ. Coauthorship networks and patterns of scientific collaboration. Proc Natl Acad Sci U S A 2004;101:5200–05. 14745042 10.1073/pnas.0307545100 PMC387296

[14] Huang T Ren X Tang X. Current perspectives and trends of CD39-CD73-eAdo/A2aR research in tumor microenvironment: a bibliometric analysis. Front Immunol 2024;15:1427380. 39188712 10.3389/fimmu.2024.1427380 PMC11345151

[15] Agarwal P Kukrele R Sharma D. Delayed revascularization of extremities following vascular injuries: challenges and outcome. J Orthop 2023;35:31–36. 36387761 10.1016/j.jor.2022.10.016 PMC9660842

[16] Farzin A Miri AK Sharifi F. 3D-printed sugar-based stents facilitating vascular anastomosis. Adv Healthc Mater 2018;7:e1800702. 30375196 10.1002/adhm.201800702 PMC6394876

[17] Liu SQ Lei P Cui XH. Sutureless anastomoses using magnetic rings in canine liver transplantation model. J Surg Res 2013;185:923–33. 23972622 10.1016/j.jss.2013.07.025

[18] Boyd LC Bond GA Hamidian Jahromi A. Microvascular reconstruction of pediatric lower extremity trauma using free tissue transfer. European J Orthop Surg Traumatol 2019;29:285–93. 30649621 10.1007/s00590-019-02367-w

[19] Lin J Wang ZJ. Replantation of severed fingers in children. In: Hou C Chang S Tang J Cai Z, eds.. Practical Microsurgery Cases: Repair, Replantation and Reconstruction. Springer Singapore; 2021:19–21.

[20] Martin RS Edwards WH Mulherin JL. Cryopreserved saphenous vein allografts for below-knee lower extremity revascularization. Ann Surg 1994;219:664–70. 8203975 10.1097/00000658-199406000-00009 PMC1243216

[21] Shaterian A Rajaii R Kanack M. Predictors of digit survival following replantation: quantitative review and meta-analysis. J Hand Microsurg 2018;10:66–73. 30154618 10.1055/s-0038-1626689 PMC6103763

[22] Sharma S Lin S Panozzo A. Thumb replantation: a retrospective review of 103 cases. Ann Plast Surg 2005;55:352–56. 16186696 10.1097/01.sap.0000181343.23091.93

[23] Tamulevicius M Steinbach MD Bucher F. Impact of single versus dual arterial supply on perfusion and function in finger replantation after complex hand injuries. Sci Rep 2025;15:3169. 39863591 10.1038/s41598-025-85525-x PMC11763008

[24] Lee BI Chung HY Kim WK. The effects of the number and ratio of repaired arteries and veins on the survival rate in digital replantation. Ann Plast Surg 2000;44:288–94. 10735221 10.1097/00000637-200044030-00007

[25] Maricevich M Carlsen B Mardini S. Upper extremity and digital replantation. Hand (N Y) 2011;6:356–63. 23204960 10.1007/s11552-011-9353-5 PMC3213257

[26] Puhaindran ME Paavilainen P Tan DMK. Dermal pocketing following distal finger replantation. J Plast Reconstr Aesthet Surg 2010;63:1318–22. 19620030 10.1016/j.bjps.2009.06.039

[27] Arata J Ishikawa K Soeda H. Replantation of fingertip amputation by palmar pocket method in children. Plast Reconstr Surg 2011;127:78e–80e. 21364399 10.1097/PRS.0b013e3182063665

[28] Purisa H Ozturk MB Kabakas F. Intramedullary venous drainage system for distal fingertip replantations. Ann Plast Surg 2017;79:166–73. 28570454 10.1097/SAP.0000000000001095

[29] Koshima I Yamashita S Sugiyama N. Successful delayed venous drainage in 16 consecutive distal phalangeal replantations. Plast Reconstr Surg 2005;115:149–54. 15622245

[30] Wang SH Hsu CC Tzou CHJ. Synchronous microsurgical anastomosis in complex replantation surgery. J Hand Surg Eur Vol 2018;43:1044–49. 30282504 10.1177/1753193418802149

[31] Sert G Sakarya AH. Safe, fast, and minimally-assisted microsurgical anastomosis with combined open-loop suturing and airborne tying: a clinical and experimental study. Ulus Travma Acil Cerrahi Derg 2023;29:449–57. 36995208 10.14744/tjtes.2023.79702 PMC10214895

[32] Win TS Murakami N Borges TJ. Longitudinal immunological characterization of the first presensitized recipient of a face transplant. JCI Insight 2017;2:e93894. 28679959 10.1172/jci.insight.93894 PMC5499367

[33] Kaufman CL Kanitakis J Weissenbacher A. Defining chronic rejection in vascularized composite allotransplantation—the American society of reconstructive transplantation and international society of vascularized composite allotransplantation chronic rejection working group: 2018 American society of reconstructive transplantation meeting report and white paper Research goals in defining chronic rejection in vascularized composite allotransplantation. SAGE Open Med 2020;8:2050312120940421. 32704373 10.1177/2050312120940421 PMC7361482

[34] Kollar B Kamat P Klein HJ. The significance of vascular alterations in acute and chronic rejection for vascularized composite allotransplantation. J Vasc Res 2019;56:163–80. 31266018 10.1159/000500958

[35] Fisher DT Mackey E Kononov E. Chronic rejection models for vascularized composite tissue allotransplantation. Sci Rep 2025;15:16882. 40374749 10.1038/s41598-025-01803-8 PMC12081738

[36] Thölking G Gerth HU Schuette-nuetgen K. Influence of tacrolimus metabolism rate on renal function after solid organ transplantation. World J Transplant 2017;7:26–33. 28280692 10.5500/wjt.v7.i1.26 PMC5324025

[37] Wyzgal J Oldakowska-jedynak U Paczek L. Posttransplantation diabetus mellitus under calcineurin inhibitor. Transplant Proc 2003;35:2216–18. 14529893 10.1016/s0041-1345(03)00819-4

[38] Brandacher G. Vascularized composite allotransplantation: a field is maturing. Organ Transplantation 2018;23:559. 30080699 10.1097/MOT.0000000000000574

[39] Jank BJ Xiong L Moser PT. Engineered composite tissue as a bioartificial limb graft. Biomaterials 2015;61:246–56. 26004237 10.1016/j.biomaterials.2015.04.051 PMC4568187

[40] Shieh SJ Cheng TC. Regeneration and repair of human digits and limbs: fact and fiction. Regeneration 2015;2:149–68. 27499873 10.1002/reg2.41 PMC4857729

[41] Chan JKK Harry L Williams G. Soft-tissue reconstruction of open fractures of the lower limb: muscle versus fasciocutaneous flaps. Plast Reconstr Surg 2012;130:284e. 10.1097/PRS.0b013e3182589e63 PMC3408595 22842425

[42] Lok E Oe T Ng S. Lower extremity traumatic wound management: relative significance of negative pressure wound therapy in the orthopedic setting. Adv Wound Care (New Rochelle) 2025;14:327–43. 39001834 10.1089/wound.2023.0133

[43] Prokuski V Strohl A. Soft tissue coverage for severe infections. Hand Clin 2020;36:369–79. 32586464 10.1016/j.hcl.2020.03.011

[44] Ranat K Phan H Ellythy S. Advancements in musculoskeletal tissue engineering: the role of melt electrowriting in 3D-printed scaffold fabrication. J Funct Biomater 2025;16:163. 40422828 10.3390/jfb16050163 PMC12112603

[45] Grasa J Urbiola A Flandes-iparraguirre M. Modeling and fabrication of MEW-3D tubular scaffolds with tendon mechanical behavior for tenocyte differentiation. Acta Biomater 2025;197:226–39. 40089128 10.1016/j.actbio.2025.03.006

[46] Burdon J Taplin S Kay SP. The functional assessment and rehabilitation programme of the UK hand and upper limb transplant service. Hand Therapy 2020;25:18–25.

[47] Young W Daya M Govender P. Functional outcome using early controlled active motion in rehabilitation of a replanted hand: a case report. J Hand Ther 2020;33:426–34. 30857892 10.1016/j.jht.2018.10.004

[48] Mavrogenis AF Spyridonos SG Antonopoulos D. Effect of sensory re-education after low median nerve complete transection and repair. J Hand Surg Am 2009;34:1210–15. 19556076 10.1016/j.jhsa.2009.04.014

[49] Che Daud AZ Yau MK Barnett F. Integration of occupation based intervention in hand injury rehabilitation: a randomized controlled trial. J Hand Ther 2016;29:30–40. 26847318 10.1016/j.jht.2015.09.004

[50] Salminger S Sturma A Roche AD. Functional and psychosocial outcomes of hand transplantation compared with prosthetic fitting in below-elbow amputees: a multicenter cohort study. PLOS ONE 2016;11:e0162507. 27589057 10.1371/journal.pone.0162507 PMC5010226

[51] Bueno A Nevado-sanchez E Collazo C. Functional outcomes in upper limb replantation—a systematic review. J Clin Med 2024;13:1289. 38592128 10.3390/jcm13051289 PMC10931822

[52] Briganti M Delnevo CD Brown L. Bibliometric analysis of electronic cigarette publications: 2003−2018. Int J Environ Res Public Health 2019;16:320. 30682767 10.3390/ijerph16030320 PMC6388121

[53] Zyoud SH. Global scientific trends on aflatoxin research during 1998-2017: a bibliometric and visualized study. J Occup Med Toxicol 2019;14:27. 31832075 10.1186/s12995-019-0248-7 PMC6873441

[54] Boudry C Baudouin C Mouriaux F. International publication trends in dry eye disease research: a bibliometric analysis. Ocul Surf 2018;16:173–79. 29031646 10.1016/j.jtos.2017.10.002

[55] Zhang XL Zheng Y Xia ML. Knowledge domain and emerging trends in vinegar research: a bibliometric review of the literature from WoSCC. Foods 2020;9:166. 32050682 10.3390/foods9020166 PMC7074530

[56] Zhao T Zhang Y Dai Z. Bibliometric and visualized analysis of scientific publications on ossification of the posterior longitudinal ligament based on web of science. World Neurosurg 2021;149:e231–e243. 33610866 10.1016/j.wneu.2021.02.045

